# Transcriptome profiles of metamorphosis in the ornamented pygmy frog *Microhyla fissipes* clarify the functions of thyroid hormone receptors in metamorphosis

**DOI:** 10.1038/srep27310

**Published:** 2016-06-02

**Authors:** Lanying Zhao, Lusha Liu, Shouhong Wang, Hongyuan Wang, Jianping Jiang

**Affiliations:** 1 Chengdu Institute of Biology, Chinese Academy of Sciences, Chengdu 610041, China; 2 University of Chinese Academy of Sciences, Beijing 100049, China; 3 College of Life Sciences, Shaanxi Normal University, Xi’an 710062, China

## Abstract

Anuran metamorphosis is an excellent system in which to study postembryonic development. Studies on *Xenopus* (Mesobatrachia) show that thyroid hormone receptors (TRs) regulate metamorphosis in a ligand-dependent manner by coordinating the action of hundreds of genes. However, whether this mechanism is conserved among amphibians is still unknown. To understand the molecular mechanism of this universal phenomenon, we report the transcriptional profiles of the three key developmental stages in *Microhyla fissipes* (Neobatrachia): premetamorphosis (PM), metamorphic climax (MC) and completion of metamorphosis (CM). In total, 2,293 differentially expressed genes were identified from comparisons of transcriptomes, and these genes showed stage-specific expression patterns. Unexpectedly, we found that TR*α* was highly expressed in *Xenopus laevis* and *Bufo gargarizans* at premetamorphosis but showed low expression in *M. fissipes*. In contrast, TR*β* was highly expressed during metamorphosis in *M. fissipes* and *X. laevis*. This result may imply that TR*β* is essential for initiating metamorphosis, at least in *M. fissipes*. Thus, our work not only identifies genes that are likely to be involved in Neobatrachia metamorphosis but also clarifies the roles of unliganded TRα in regulating tadpole growth and timing of metamorphosis, which may be conserved in anurans, and the role of liganded TRβ in launching metamorphosis.

Metamorphosis is a postembryonic developmental process in which animals reshape and restructure their body’s morphology, anatomy and even physiology due to cell proliferation, programmed cell death, cell differentiation, and tissue remodeling^1^. It is an important developmental transition that occurs in many animals, such as some insects, fishes, amphibians, mollusks, crustaceans, cnidarians, echinoderms, and tunicates^2^,^3^,^4^.

Metamorphosis especially in anurans of amphibian is an exquisite model for postembryonic development in vertebrates^5^,^6^,^7^ and thyroid hormones (THs) play essential roles in regulating metamorphosis in anurans. Studies on anuran metamorphosis are mainly conducted in *Xenopus* (Mesobatrachia). Based on the concentrations of endogenous TH and the morphological characters, *Xenopus* metamorphosis can be divided into 3 main stages^8^,^9^: premetamorphosis (PM), prometamorphosis (POM) and metamorphic climax (MC). In PM, when TH is absent, the unliganded thyroid hormone receptor/9-cis-retinoic acid receptor (TR/RXR) heterodimers recruit corepressors to repress downstream genes and prevent premature metamorphic changes^10^. TH becomes available during POM, and its level throughout the body increases and peaks at the MC stage^11^. Once liganded with TH, the TR/RXR heterodimers recruit coactivators to activate the same downstream genes, leading to metamorphic changes^10^,^12^,^13^. This extensive process is mainly characterized by organ resorption, tissue morphogenesis and tissue remodeling^8^. Subsequently, endogenous TH levels drop to a low but measurable concentration at the completion of metamorphosis (CM)^14^. Thus, TRs/RXRs, as switches, regulate *Xenopus* metamorphosis in a ligand-dependent manner by coordinating the action of hundreds of genes. These genes are associated with many cellular processes, including cell proliferation, programmed cell death and cell differentiation.

*Xenopus laevis* has two TRs, TRα and TRβ, to mediate the effects of TH on the genes that it regulates^10^,^15^. TRα-deficient tadpoles develop faster, and their body size is much smaller than that of their wild-type siblings, suggesting that TRα plays important roles in controlling the timing of metamorphosis^16^,^17^. Although TRα has been reported as an essential factor for metamorphosis initiation^10^,^15^, metamorphosis still occurred in TR*α*-deficient *X. laevis*. It remains unknown whether TR*β* or other unknown thyroid hormone receptors are essential for metamorphosis initiation.

Because *Xenopus* is only a representative of Mesobatrachia, it is necessary to choose another species, ideally one from the Neobatrachia, to investigate whether the molecular mechanism of metamorphosis regulation is conserved in other anuran species. In addition, given the role of TRα in regulating developmental rate and body size in *Xenopus*, for this study, we chose the Neobatrachian *M. fissipes* because it has a much smaller body size and shorter timing of metamorphosis than *Xenopus*^18^. RNA-Seq analysis was performed on *M. fissipes* samples from 3 key developmental stages (PM, MC, and CM), and then the transcriptome profiles were constructed. Comparison of the transcriptomes identified a total of 2,293 differentially expressed genes, which were then clustered using hierarchical clustering. We performed GO enrichment analysis to identify biological processes in the progression of metamorphosis. We also clustered differentially expressed genes using a self-organizing map (SOM), and genes showing stage-specific expression patterns and that may play important roles in specific stages were identified. Notably, the expression level of TR*α*, which is highly expressed in *X. laevis*, *Rana nigromaculata*^19^ and *B. gargarizans* at premetamorphosis, was expressed at low levels in *M. fissipes* in all three stages. Furthermore, we found that TR*β* was highly expressed at the metamorphic climax stage of *M. fissipes*, as it is in *X. laevis*^15^, *R. nigromaculata*^19^, *Pelobates cultripes*, *Scaphiopus couchii* and *Spea multiplicata*^20^. Together with recent work in TR*α*-deficient *Xenopus tropicalis*, our work not only provides genes that are likely involved in Neobatrachia metamorphosis but also provides further evidence implying that TRα is critical for regulating tadpole growth and the timing of metamorphosis. Meanwhile, our data also imply that the liganded TRβ may be important for launching metamorphosis.

## Results

### *De novo* transcriptome assembly of *Microhyla fissipes* and transcriptome annotation

**Figure 1:**
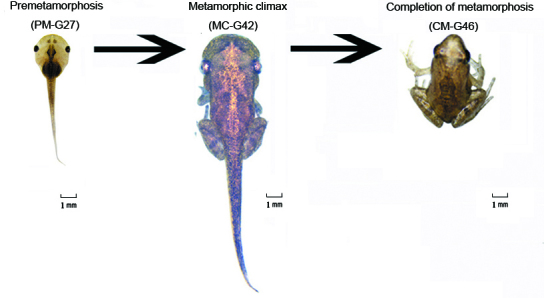
Samples of the three developmental stages used for gene expression profile analysis. *Microhyla fissipes* in the premetamorphosis (PM), metamorphic climax (MC) and completion of metamorphosis (CM) stages are presented at the left, middle and right, respectively. The scale of the animal’s body size is 1:5, and the scale bar is 1 mm.

We sequenced the genome-wide transcriptomes of three stages of *M. fissipes*, PM, MC and CM (Fig. 1), and obtained totals of 55,656,754, 59,588,250 and 62,400,210 paired-end raw reads, respectively. All of these Illumina paired-end reads have been submitted to the NCBI short read archive (SRA). The Q30 of the 3 samples was more than 90%. After removal of the adaptor sequences and ambiguous or low-quality reads, we obtained a total of 177,585,214 (94.09% of the raw reads) clean reads from the three stages. These reads were then *de novo* assembled, generating 110,061 assembled unigenes which had an average length of 732 bp and an N50 of 1,330 bp (Table 1).

To annotate these assembled unigenes, we next carried out BLASTX searches against the NCBI non-redundant protein database (Nr), the NCBI non-redundant nucleotide database (Nt), the Swiss-Prot and euKaryotic Ortholog Groups (KOG), the Gene Ontology (GO) and the Kyoto Encyclopedia of Genes and Genomes Ortholog (KO) databases. In total, 27,267, 12,905, 23,293, 13,459, 26,692 and 12,134 unigenes were annotated based on these databases, respectively (Table 1). The most unigenes were annotated to the Nr database. The E-value distribution showed that 60.07% of the annotated sequences had strong homology (E-value below 1E-50), and the similarity distribution showed that 85.61% of the annotated sequences had a similarity greater than 60% (Supplementary Table S1 and Supplementary Fig. S1). In addition, functional classification was also performed against the Gene Ontology (GO) and Kyoto Encyclopedia of Genes and Genomes (KEGG) databases. Based on the GO database, 26,692 unigenes were assigned to 51 level-2 GO terms, which were summarized under three main GO categories, cellular component, molecular function and biological process (Supplementary Fig. S2). Within the GO categories cellular component, molecular function and biological process, 17, 12 and 22 level-2 categories, respectively, were identified. KEGG was used to identify potential biological pathways, and 12,134 unigenes were assigned to 268 KEGG pathways (Supplementary Fig. S3). In total, 34,938 unigenes (31.74% of the 110,061 unigenes) were annotated in at least one database.

### Protein coding sequence (CDS) prediction

To extract coding regions from the assembled unigenes, unigenes were first aligned to protein databases in the priority order NR and then Swiss-Prot. Unigenes aligned to a high-priority database were not aligned to a database of lower priority. The process ended when all alignments had been performed. The correct reading frame of the nucleotide sequences of unigenes (5′-3′ direction) was defined by the highest rank in the BLAST results, and the corresponding protein sequences were obtained from the standard codon table. In total, 26,323 unigenes were found to contain a CDS. The size-frequency distributions of the CDS and proteins of these unigenes are shown in Supplementary Fig. S4. Unigenes that could not be aligned to any database were scanned with ESTScan^21^ to predict possible CDS. We obtained 51,506 CDS in 51,420 unigenes. The majority of the unigene CDS (92.72%) assigned by ESTScan were shorter than 500 bp (Supplementary Fig. S4). The protein sequences were obtained from ESTScan as above (Supplementary Fig. S4). In total, 77,743 unigenes were potential protein-coding genes, comprising 70.636% of the total unigenes.

### Global outlook on the gene expression levels during *Microhyla fissipes* metamorphosis

**Figure 2:**
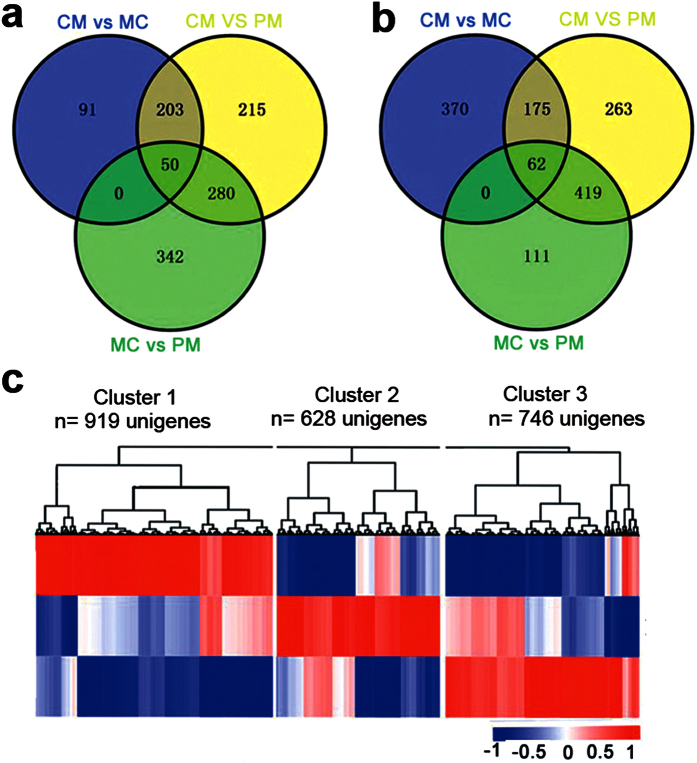
Analysis of differentially expressed unigenes during *Microhyla fissipes* metamorphosis. Venn diagrams show the number of genes significantly **(a)** up-regulated and **(b)** down-regulated; green, blue and yellow circles represent the changes from PM to MC, MC to CM, and PM to CM, respectively. **(c)** Similarity of the expression profiles between genes with hierarchical clustering is shown above the heatmap. Intensity of color indicates expression levels, and the three major clusters represent the PM, MC and CM stages.

**Figure 3:**
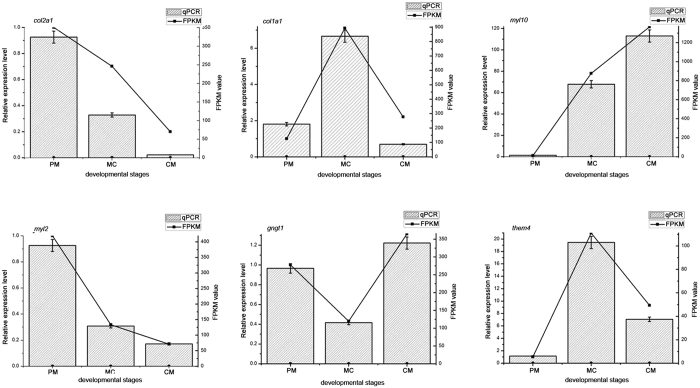
Expression profiles of six genes. The X axis represents the developmental stages. Lines represent the FPKM value of the transcriptome result (Y axis at right). Columns and bars represent the means and standard errors of three individual samples (Y axis at left). Each experiment was performed in triplicate.

To obtain a perspective on global gene expression levels in the three key developmental stages of metamorphosis, we performed pairwise comparisons (MC vs. PM, CM vs. MC, and CM vs. PM). In all, 1,181 and 1,400 unigenes (Fig. 2a,b) were significantly up- and down-regulated, respectively (Supplementary Table S2), and these differentially expressed gene sets were combined into one set. We found 2,293 unigenes that were differentially expressed in the three key developmental stages. Among these unigenes, 50 and 62 unigenes were continuously up-regulated and down-regulated, respectively, from the PM stage to the CM stage. To validate the RNA-Seq results, six genes (*col2a1*, *cola1*, *myl10, myl2, gngt1* and *them4*) were randomly selected for further confirmation by real-time PCR. The gene *rpl37* was chosen as the internal control because Rpl37 is a ribosomal protein and its expression level is relatively stable^22^, which was supported by our RNA-Seq result. The real-time PCR results of the six chosen genes were consistent with the RNA-Seq expression profiles (Fig. 3).

To identify molecular pathways involved in the transformation from tadpole to froglet, the 2,293 differentially expressed unigenes were grouped into three major clusters (Supplementary Table S3 and Fig. 2c). These clusters contained 919, 628 and 746 unigenes that were expressed mostly in the PM, MC and CM stages, respectively, indicating that these genes played vital roles in the biological processes of different stages.

**Figure 4:**
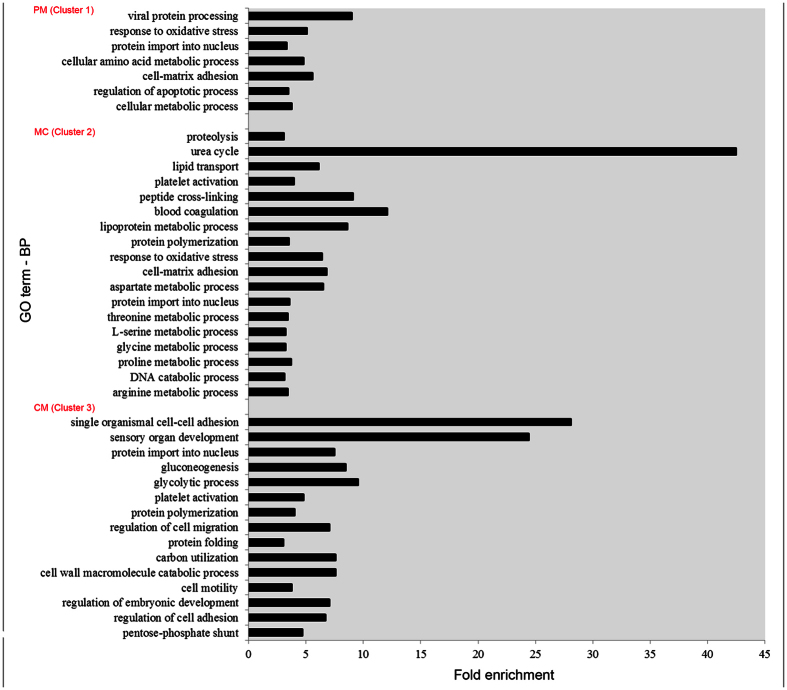
GO enriched processes in three key developmental stages. The significantly overrepresented (FDR < 0.05) enrichment factors of GO biological processes of the three clusters using hierarchical clustering are shown (Table S7). Only groups containing more than five genes and having a fold enrichment factor >3 are presented.

To understand the biological pathways of the genes in the three identified expression clusters, we used GO enrichment analysis to identify biological process categories that were statistically enriched in each cluster with reference tothe assembled unigenes. The analysis revealed some overlaps in the GO categories (Supplementary Table S4 and Fig. 4). Enrichment of response to oxidative stress (GO:0006979) in cluster 1 and cluster 2 indicates a higher oxygen-binding rate of tadpole hemoglobin than froglet hemoglobin. In cluster 1, regulation of apoptotic process (GO:0042981) was enriched, which is consistent with apoptosis as an early event during metamorphosis^23^. In cluster 2, 18 GO categories were enriched. These categories included proteolysis (GO:0006508), urea cycle (GO:0000050), DNA catabolic process (GO:0006308) and the metabolism of some amino acids, among others. In cluster 3, 15 GO categories were enriched. These included sensory organ development (GO:0007423), regulation of cell migration (GO:0030334), cell motility (GO:0048870), and regulation of embryonic development (GO:0045995). Interestingly, cell-matrix adhesion (GO:0007160) was enriched in clusters 1 and 2, and single organismal cell-cell adhesion (GO:0016337) and regulation of cell adhesion (GO:0030155) were enriched in cluster 3. This is consistent with the previous observation that cell-cell and cell-ECM interactions are involved in metamorphosis^24^,^25^. The modification of the ECM is expected to alter cell-cell and cell-ECM interactions, thus affecting cellular changes including apoptosis, cell proliferation, migration and differentiation (Fig. 4 and Supplementary Table S4).

### Stage-specific expression patterns of differentially expressed genes across developmental stages

**Figure 5:**
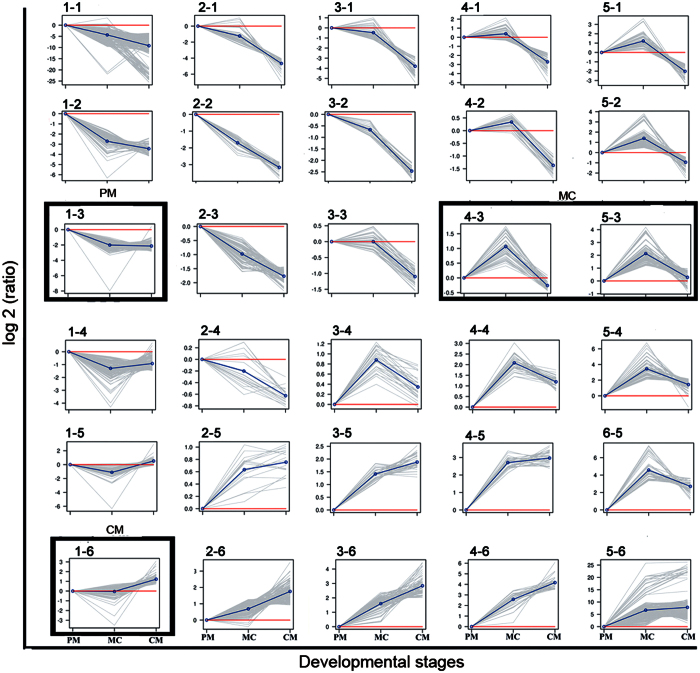
Clustering profiles. Plots of SOM profiles of the reduced set with their centroids highlighted in blue. Four clusters that were further analyzed for stage-specific expression patterns are marked in black boxes. The vertical axis represents the relative gene abundance, and the horizontal axis shows the three developmental stages.

To further identify genes with roles that vary in importance across developmental stages, expression patterns were deduced according to the fold changes of expression levels in the MC and CM stages compared with that in the PM stage using SOM clustering. In this way, 30 expression patterns were deduced (Fig. 5, Supplementary Table S5). We found 4 clusters that showed stage-specific high expression in one of the three developmental stages (Fig. 5). Unigenes of the 1-3 cluster were specifically expressed in the PM stage. While unigenes of the 4-3 and 5-3 clusters were specifically expressed in the MC stage, unigenes of the 1-6 cluster were specifically expressed in the CM stage. In the PM-specific high-expression cluster (Supplementary Table S6), we found 132 putative stage-specific unigenes, including genes that regulate transcription, such as *c-myc* and *c-fos*. In the MC-specific high-expression cluster (Supplementary Table S7), we observed 122 putative stage-specific unigenes. Genes belonging to the ECM were included, and most of these ECM genes were collagen genes, including the fibrillar forms *col1a1, col1a2, col3a1*, and *col5a1* and the non-fibrillar forms *col6a1, col6a2, col6a3*, and *col18a1*. In addition, genes related to ECM proteolysis were also included, such as matrix metalloproteinases (MMPs) and a disintegrin and metalloproteinase (ADAM). We also found the hedgehog target gene *twist2* among the MC pattern genes. In the CM-specific high-expression cluster (Supplementary Table S8), we observed 135 putative stage-specific unigenes. GO enrichment analysis at the CM stage showed that a majority of enriched GO categories were associated with metabolic and catabolic processes (Supplementary Table S9). This result is consistent with the fact that the animal starts to feed again at the end of metamorphosis. In addition, we found that the tumor suppressor candidate genes kruppel-like factor 4 (*klf4*) and *myo18b* were up-regulated in this cluster.

### The expression profiles of genes related to the initiation of metamorphosis

**Figure 6:**
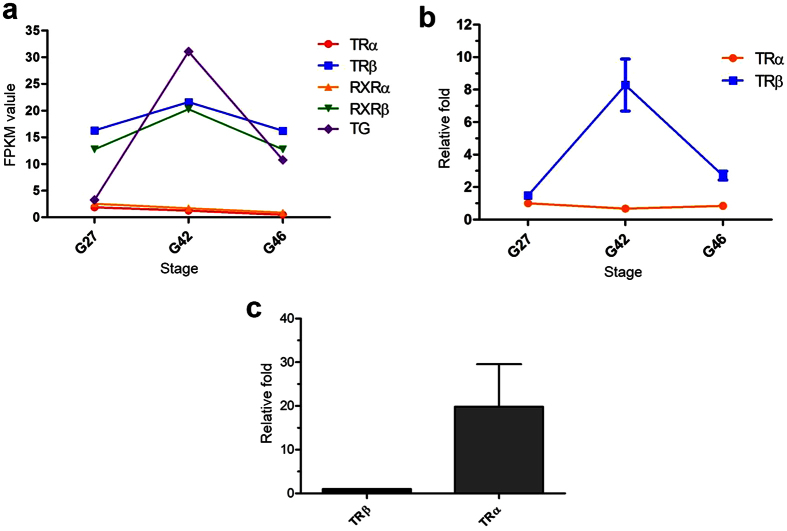
Expressional profiles of thyroid hormone (TH) - related genes involved in regulating metamorphosis. (**a**) Correlation of the expression levels of TR*α*, RXR*α*, TR*β*, RXR*β* and *TG* mRNAs with the three key developmental stages of *M. fissipes*. The vertical axis represents expression levels (FPKM value), and the horizontal axis represents developmental stages. (**b**) Expression profiles of TR genes in three key developmental stages of *M. fissipes*. The X axis represents the developmental stages. Lines and bars represent the means and standard errors of three individual samples (Y axis). Each experiment was performed in triplicate. (**c**) Comparison of the expression levels of TR genes in *B. gargarizans* at the PM stage. The X axis represents two TR genes. Columns and bars represent the means and standard error of three individual samples. Each experiment was performed in triplicate.

The TR/RXR heterodimer plays vital roles in sensing TH and regulating metamorphosis. In all, based on the annotation results, we detected five genes (TR*α*, TR*β*, RXR*α*, RXR*β*, thyroglobulin (TG)) that were involved in initiating the process of metamorphosis. As shown in Fig. 6a, the expression level of *TG*, in parallel with TR*β* and RXR*β* mRNA, increased and reached a peak in the MC stage and then declined in the CM stage to the same level as in the PM stage. Interestingly, we noted that the expression level of TR*β* was higher than that of TR*α* in *M. fissipes*. To further validate this result, we measured the expression levels of TR*α* and TR*β* in *M. fissipes* samples from the three different stages using absolute real-time quantitative PCR. The results showed that the expression level of TR*β* was higher than that of TR*α* in *M. fissipes* at all three stages (Fig. 6b). In addition, we also measured the expression levels of TR*α* and TR*β* in *Bufo gargarizans* at Gosner stage 27 by relative real-time quantitative PCR (Fig. 6c). The results showed that, unlike in *M. fissipes*, the expression level of TR*α* was higher than that of TR*β* at premetamorphosis in *X. laevis*^15^ and *B. gargarizans*.

### The timing of metamorphosis and the regulation of body length in three anuran species

We also measured the metamorphosis timing and body size of three anuran species (*M. fissipes*, *X. laevis* and *B. gargarizans*) that were reared under identical laboratory conditions (20 °C). It took only 21 days for *M. fissipes* to reach Gosner stage 33 (metamorphosis onset). However, *B. gargarizans* and *X. laevis* took 29 and 30 days, respectively, to attain metamorphosis onset (Supplementary Table S10). Meanwhile, at the onset of metamorphosis, *M. fissipes* had the shortest body length (17.973 ± 0.344 mm), with *B. gargarizans* and *X. laevis* being longer, at 21.22 ± 0.384 mm and 35.956 ± 0.534 mm, respectively (Supplementary Table S10). Correspondingly, the mean growth rate from stage 19 to stage 33 was 0.735 mm/day in *M. fissipes* and 0.592 mm/day in *B. gargarizans*, respectively.

## Discussion

Here, we report the transcriptome analysis of *M. fissipes* using the Trinity platform. In this study, comparative expression profiling was performed for the three key developmental stages during *M. fissipes* metamorphosis. This is the first transcriptome analysis spanning the postembryonic process of *M. fissipes*. Our findings not only demonstrate that each stage in the life cycle of *M. fissipes* has a unique expression profile that is involved in particular developmental events but also clarify the functions of TRs (TRα and TRβ) in metamorphosis.

PM refers to the stage that includes early tadpole growth and development. The high expression of the proto-oncogenes *c-myc* and *c-fos* (Supplementary Table S6) suggests that the larval cells are active in the PM stage. *c-fos* and *c-myc* are involved in various processes, including apoptosis and cell proliferation^26^,^27^. In addition, this result suggests that cell activity is activated in the PM stage, which is consistent with the observation that tadpoles undergo rapid growth in the PM stage^8^. These results provide molecular evidence of the rapid growth of the tadpole body.

Notably, in the MC stage, anurans commonly undergo rapid morphological changes due to the highest endogenous TH levels. Our results show that collagen mRNAs are relatively up-regulated in the MC stage (Supplementary Table S7). Previous studies have found that branching morphogenesis of human mammary epithelial cells is enhanced in collagen I gels^28^. This result indicates that the increasing collagens may accelerate morphogenesis in the MC stage and provides molecular evidence of rapid morphological changes in the MC stage. During MC, most metamorphic changes can be described as profound remodeling of the existing organs, including the liver^29^, skin^30^ and trunk muscle^31^. Tissue remodeling is an important function of matrix metalloproteinases (MMPs)^32^. Among the MMP family members, MMP14 (also known as membrane type 1 MMP; MT1 MMP), MMP19 and TIMP2 were detected in our study. Previous studies showed that MMP14 is associated with TIMP2 at the cell surface^33^ and promotes vascular remodeling^34^. In addition, adamalysin proteins cleave ECM components to influence tissue remodeling^35^,^36^. Two adamalysin proteins, ADAM19 and ADAMTS7, also showed an MC-specific expression pattern (Supplementary Table S7). Taken together, these results suggest that genes associated with ECM remodeling play a vital role in *M. fissipes* tissue remodeling in the MC stage. In addition, the evolutionarily conserved hedgehog pathway plays very important roles in the process of development. A hedgehog target gene *twist2*, which is reported to promote the epithelial-to-mesenchymal transition (EMT)^37^, is up-regulated in the MC stage, which suggests that the hedgehog pathway participates in the remodeling of the existing organs in the MC stage.

In the CM stage, we found high expression levels of *myo18b* and *klf4*, which are associated with negative regulation of the cell cycle (Supplementary Table S8). MYO18B, a class XVIII myosin protein, is a tumor suppressor^38^. In addition, *klf4* is a growth arrest-associated gene that inhibits cell proliferation^39^,^40^. These data suggest that cell proliferation and differentiation are suppressed in the CM stage, which is consistent with the observation that cell proliferation and differentiation are inactive in froglets. Our results provide molecular evidence that the transition from tadpoles to froglets is almost completed in the CM stage.

TH plays a causative role in amphibian metamorphosis^11^,^41^,^42^. It is generally believed that the regulatory function of TH is mediated by TR/RXR heterodimers^43^,^44^. *M. fissipes* has two TR (TR*α* and TR*β*) and two RXR (RXR*α* and RXR*β*) genes. We found that in *M. fissipes*, the premetamorphosis expression level of TR*α* is lower than that of TR*β* (Fig. 6a,b), in contrast to their relative expression levels in *Xenopus*^15^ and *B. gargarizans* (Fig. 6c). Meanwhile, *M. fissipes* spends shorter time to attain the onset of metamorphosis and has a smaller body size than either *Xenopus* or *B. gargarizans* (Supplementary Table S10). Furthermore, *M. fissipes* also shows a higher growth rate than that of *B. gargarizans*, which supports the hypothesis that unliganded TRα inhibits growth rate in both *M. fissipes* and *B. gargarizans*. These results are consistent with early studies showing that unliganded TRα is expected to repress metamorphic genes^45^,^46^. Species with low expression of TRα before TH becomes available could show less repression of metamorphic genes and a faster onset of metamorphosis, as seen in TRα-mutant experiments in *Xenopus*^16^,^17^. TRα-deficient tadpoles develop faster and are much smaller in body size than their wild-type siblings. Body size and the timing of metamorphosis are determined by both genetic and environmental factors. Animals in our study were reared under laboratory conditions, which can eliminate the environmental effects. Thus, the fast development and small body size in *M. fissipes* are attributed to the effects of the low expression of TRα. It seems clear that TRα plays a role in determining the growth rate and the body size. Our study further underscores the importance of TRα in anuran metamorphosis. In addition, TRα knockdown tadpoles have also delayed natural metamorphosis^16^. However, in our study, it takes longer time to undergo the period of prometamorphosis in *M. fissipes* than in *B. gargarizans* and *X. laevis*. These data could not lead to a conclusion that the liganded TRα has function in accelerating development in *M. fissipes* as it in *X. laevis*.

It is worth noting that although the expression level of TRα is very low in all three developmental stages in *M. fissipes* (Fig. 6a,b), the metamorphic changes still take place. This observation raises the interesting speculation that TRβ is essential for initiating metamorphosis by activating TH-responsive genes. Firstly, recent work showed that metamorphosis still occurred in TRα mutant *X. tropicalis* tadpoles^16^,^17^. Secondly, our data show that the expression level of TR*α* is very low during metamorphosis in *M. fissipes* (Fig. 6a,b). Thirdly, in *X. laevis*^15^, *R. nigromaculata*^19^, *P. cultripes*, *S. couchii*, *S. multiplicata*^20^, *M. fissipes* and even TR*α* mutant *X. tropicalis*^16^,^17^, TRβ is still highly expressed during metamorphosis. Last but not least, the TH receptor TR*β* is an important regulator of metamorphosis in *Xenopus*^15^. Thus, our data suggest that TRβ is essential for metamorphosis, at least in *M. fissipes*.

## Methods

### Sample preparation

*Microhyla fissipes* were collected from paddy fields in Chengdu (Sichuan, China; 103°43′35″ E, 30°41′28″ N, 541.5 m) in May 2014. We raised the animals in the laboratory and applied artificial breeding. We obtained a cluster of tadpoles with the same genetic background. Based on the staging method for anurans^47^ and using morphological characters, samples were collected in Gosner 27, Gosner 42 and Gosner 46 (Fig. 1), representing the three key developmental periods of PM, MC and CM, respectively. We snap froze samples in liquid nitrogen for 2 hours and then stored them at −80 °C. To minimize biological variance, nine tadpoles were sacrificed, using three individuals from each of the three stages as independent biological replicates. Animal materials were collected legally. The experimental procedures were approved by the Animal Use Ethics Committee of the Chengdu Institute of Biology. All animal collection and use protocols were carried out in accordance with the appropriate guidelines and regulations.

Total RNA of nine samples was extracted separately using Unizol Reagent (Biostar, China) following the manufacturer’s instructions. After RNA purification, concentration and integrity were quantified, and equal amounts of total RNA from the three samples from the same stage were pooled for library construction and sequencing.

### cDNA library construction and Illumina sequencing

The mRNAs were purified separately from the 3 RNA samples using poly-T oligo-attached magnetic beads. The mRNAs were subsequently fragmented. First-strand cDNA was synthesized using random hexamer primers and M-MuLV Reverse Transcriptase (RNase H^−^). Second-strand cDNA synthesis was subsequently performed using DNA Polymerase I and RNase H. The remaining overhangs were converted into blunt ends via exonuclease/polymerase activities. After adenylation of the 3′ ends of the DNA fragments, NEBNext Adaptors with a hairpin loop structure were ligated to prepare for hybridization. To preferentially select cDNA fragments of 150–200 bp in length, the library fragments were purified with AMPure XP system (Beckman Coulter, Beverly, USA). Then, 3 μl of USER Enzyme (NEB, USA) was used with size-selected, adaptor-ligated cDNA at 37 °C for 15 min followed by 5 min at 95 °C before PCR. Then, PCR was performed with a Phusion High-Fidelity DNA polymerase, universal PCR primers and Index (X) primer. Finally, PCR products were purified (AMPure XP system), and library quality was assessed on the Agilent Bioanalyzer 2100 system. The clustering of the index-coded samples was performed on a cBot Cluster Generation System using the TruSeq PE Cluster Kit v3-cBot-HS (Illumina) according to the manufacturer’s instructions. After cluster generation, the library preparations were sequenced on an Illumina HiSeq 2000 platform by NovoGene (Beijing), and paired-end reads were generated.

### *De novo* transcriptome assembly and transcriptome annotation

The library quality was verified using FastQC (version 0.10.0) software^48^. Clean reads were produced by removing reads containing adapters, reads containing poly-N and low quality reads from the raw reads. The clean reads of three libraries were assembled using Trinity^49^ as a reference transcriptome. To annotate the assembled genes of *M. fissipes*, Unigenes were first aligned with BLASTX^50^ to the databases NR, NT and Swiss-Prot with an E-value threshold of 1.0E-5 and to KOG with an E-value threshold of 1.0E-3 to predict the function of genes. Using NR annotation, Blast2GO^51^ software was used to obtain GO annotations defined by molecular function, cellular component and biological process ontologies. Pathway assignments were determined based on the KEGG database using BLASTX with an E-value threshold of 1.0E-5.

### Differential gene expression analysis and clustering

For each sequenced library, the read counts were adjusted using the edgeR program package through one scaling normalized factor. Gene expression levels for each sample were estimated using RSEM^52^. To obtain a perspective on global gene expression changes during metamorphosis, we performed pairwise comparisons (MC vs. PM, CM vs. MC, and CM vs. PM) using the DEGseq (2010) R package^53^ for screening differentially expressed genes. Unigenes were considered as differentially expressed at a normalized fold change >±2 at *q* value < 0.005 after adjustment for the false discovery rate (FDR)^54^. For better visualization, Venn diagrams show the numbers of unigenes that were significantly up- and down-regulated by pairwise comparisons.

To compare the unigene expression levels, each unigene was further normalized by fragments per kilobase of exon model per million mapped reads (FPKM) for the three developmental stages^55^. For heatmap generation, log_10_^(FPKM+1)^ values were used for each tested unigene. In addition, the differentially expressed genes were also used to deduce expression patterns using SOM (Self Organization Map) clustering analysis based on the log_2_^(ratios)^ value (ratios: FPKM of MC and CM were divided by PM, which is considered to represent the initial state)^56^.

### Quantitative real time PCR

Samples (three stages of *M. fissipes* (Gosner 27, Gosner 42, Gosner 46) and one stage of *B. gargarizans* (Gosner 27)) were used for qRT-PCR. Oligonucleotide primers used for qRT-PCR amplification are listed in Supplementary Table S11. All samples were analyzed in triplicate, and the mean values of these triplicate measurements were used for the calculations of the mRNA levels. The expression patterns of six genes (Gene IDs: comp111682_c3, comp93675_c2, comp47713_c0, comp94949_c0, comp105529_c0, comp47657_c0, comp126565_c0) in *M. fissipes* and the expression levels of TR*α*, TR*β* in *B. gargarizans* were analyzed using qRT-PCR. A HiScriptTM Q RT SuperMix for qPCR (Vazyme, Nanjing, China) was used to synthesize the cDNAs, and real-time quantification was performed using an Applied Biosystems 7300 Real Time PCR System and the AceQTM qPCR SYBR® Green Master Mix (Vazyme, Nanjing, China). PCR was performed using a program of 95 °C for 5 min, followed by 40 cycles of 95 °C for 10 s and 60 °C for 30 s. Expression levels of genes in *M. fissipes* were normalized to that of *rpl37*. The relative expression levels of genes in *M. fissipes* are presented as 2^−ΔΔCT^ values because all the genes tested show highly similar amplification efficiency. The linear regression equations and R^2^ of TR*α* and TR*β* in *B. gargarizans* are shown in Supplementary Fig. S5, and The relative expression of TR*α* and TR*β* in *B. gargarizans* was obtained as N_0_(α)/N_0_(β) = (1 + E_(β)_)^Ct(β)^/(1 + E_(α)_)^Ct(α)^ which was derived from the basic equation (N_t_ = N_0_ (1 + E)^Ct^) that describes the exponential amplification of PCR, where Ct is the number of thermocycles, E is amplification efficiency, N_t_ is the number of amplicon molecules and N_0_ is the initial number of target molecules^57^. To accurately measure the expression levels of TR*α* and TR*β* in *M. fissipes*, we performed absolute quantitative real-time PCR. An equal amount of *M. fissipes* cDNA was used as template to specifically amplify TRα or TRβ. Sequences for the PCR primers are as follows: TRα (sense: AGAATCTTAGCGGGCTGGAC; antisense: AAGAGCTCTGTGGGGCACT), TR*β* (sense: TCAGGGTACATCCCGAGCTA; antisense: CCTCAAACACTTCTAAGAACAGTGG). The PCR products were purified using a gel extraction kit (TIANGEN BIOTECH, Beijing). After confirming the PCR products by sequencing, the accurate concentrations of PCR products were measured using the A260 in a NanoDrop ND1000. Molar concentrations of the template DNA were converted by calculating their molecular weight (TRα, 752,740; TRβ, 688,034). To build standard curves, the quantified PCR products were 10-fold serially diluted and were used as qPCR templates. Standard curves were built in Graph Pad Prism software, and the linear regression equations and R^2^ were generated in Excel (Fig. S6). The real-time PCR amplification efficiency is calculated as Eff. = [10^(−1/slope)^] − 1. The quantification of TR*α* and TR*β* cDNA was carried out using real-time PCR as described above. The absolute copy number of TRα was obtained as Nα = 2.34*10^(Ct-15.88)/−3.409^, and the absolute copy number of TRβ was obtained as Nβ = 10.61*10^(Ct-14.37)/−3.502^. All the obtained copy numbers of TR*α* and TRβ in different samples were then normalized to TR*α* in Gosner stage 27 by using *rpl37* as an endogenous control.

### GO enrichment analysis

The GO database covers three domains: cellular component, molecular function and biological process (http://geneontology.org/)^58^. The Blast2GO tool was used to perform GO enrichment for unigenes that showed a different expression during three developmental stages. We used the annotation of the assembled unigenes as a background for the contingency table of the Fisher’s exact test. Overrepresented GO terms with a false discovery rate (FDR) < 0.05 were reported. GO terms in the biological process category were filtered to identify the most representative processes. Only groups containing more than five genes and having a fold enrichment factor >3 are presented (Fig. 4).

### The observation of body length and developmental timing in three anuran species

Animals were reared under identical laboratory conditions. To document the developmental time of each stage during metamorphosis, the timing at which 4/5 of the population of tadpoles changed in terms of external morphological characters was defined as the developmental time of the corresponding stage (Supplementary Table S10). For the description of metamorphosis initiation, when hind limbs begin to undergo toe differentiation, seven tadpoles of each species were sampled at random. Each larva was placed in a shallow dish filled with water, cooled until motionless by adding a piece of ice to the water, and measured for the total length (TOL). The growth rate can be estimated by the formula of (body length (stage 33)-body length (stage 19))/(time (stage 33)-time (stage 19)).

## Additional Information

**Accession codes:** Raw sequence reads can be found in the SRA database under BioProject PRJNA295354. This Transcriptome Shotgun Assembly project has been deposited at DDBJ/EMBL/GenBank under the accession GECV00000000. The version described in this paper is the first version, GECV01000000.

**How to cite this article**: Zhao, L. *et al.* Transcriptome profiles of metamorphosis in the ornamented pygmy frog *Microhyla fissipes* clarify the functions of thyroid hormone receptors in metamorphosis. *Sci. Rep.* **6**, 27310; doi: 10.1038/srep27310 (2016).

## Supplementary Material

Supplementary Information

Supplementary Dataset 1

Supplementary Dataset 2

Supplementary Dataset 3

Supplementary Dataset 4

Supplementary Dataset 5

Supplementary Dataset 6

Supplementary Dataset 7

Supplementary Dataset 8

Supplementary Dataset 9

Supplementary Dataset 10

Supplementary Dataset 11

## Figures and Tables

**Table 1:** Statistics for RNA-Seq based sequencing, assembly and functional annotation for *Microhyla fissipes*.

Sequencing results	Number of total raw reads	184,817,024
Assembling results	Number of total clean reads	177,585,214
	Number of unigenes	110,061
	Total length (nt) of total unigenes	80,600,119
	Mean length (nt) of total unigenes	732
	N50 (nt) of total unigenes	1330
Annotation	Unigenes with Nr	27,267 (24.77% of 110,061 unigenes)
	Unigenes with Nt	12,905 (11.72%)
	Unigenes with Swiss-Prot	23,293 (21.16%)
	Unigenes with KOG	13,459 (12.22%)
	Unigenes with PFAM	25,035 (22.74%)
	Unigenes with GO	26,692 (24.25%)
	Unigenes with KEGG	12,134 (11.02%)
	Total unigenes annotated	34,938 (31.74% of 110,061 unigenes)
