# Supplementary material for: Transcriptome profiles of metamorphosis in the ornamented pygmy frog Microhyla fissipes clarify the functions of thyroid hormone receptors in metamorphosis
Source: Sci Rep. 2016 Jun 2;6:27310. doi: 10.1038/srep27310 (PMC4890586; doi:10.1038/srep27310)
Supplement: Supplementary Information [file srep27310-s1.doc]

# Transcriptome profiles of metamorphosis in the ornamented pygmy frog *Microhyla fissipes* clarify the functions of thyroid hormone receptors in metamorphosis

Lanying Zhao ^1, 2^, Lusha Liu ^1^, Shouhong Wang ^1, 2^, Hongyuan Wang ^3^, Jianping Jiang ^1 *^

1. *Chengdu Institute of Biology**, Chinese Academy of Sciences, Chengdu 610041, China*

2. *University of Chinese Academy of Sciences, Beijing 100049, China*

3. *College of Life Sciences, Shaanxi Normal University, Xi’an 710062, China*

## Supplementary Information

**Figure S1:** **Characteristics of assembled unigenes annotation against the Nr dataset.** E-value and similarity distribution of BLASTX hits for unigene with a cut-off E-value of 1E-5.

**Figure S2: Distribution of Gene Ontology (GO) categories (level 2) of unigenes for *M. fissipes*.** Based on high-score BLASTX matches in the NR database, *M. fissipes* unigenes were classified into three main GO categories and 46 sub-categories. The left y-axis indicates the percentage of a specific category of genes in each main category. The right y-axis indicates the number of genes in the same category. In total, we assigned 33,475 unigenes with BLASTX matches to known proteins.

**Figure S3: Distribution of *M. fissipes* unigenes among Kyoto Encyclopedia of Genes and Genomes (KEGG) pathways.** Analysis was performed using Blast2GO and the KEGG database.

**Figure S4: *M. fissipes* transcriptome coding sequences (CDS) predicted by BLASTX and ESTScan software.** (A) Length frequency distribution of predicted CDS. (B) Length frequency distribution of predicted protein sequences. (C) Length frequency distribution of CDS predicted using ESTScan software. (D) Length frequency distribution of protein sequences predicted using ESTScan software.

**Figure S5: The standard curves of TR*α* and TR*β* in** ***B. gargarizans*.** The cDNA of *B. gargarizans* at Gosner 27 was 10-fold serially diluted and used as qPCR templates. The linear regression equations and R^2^ are shown.

**Figure S6: The standard curves of TR*α* and TR*β* in *M.fissipes*.** The quantified PCR products of TRα and TRβ were 10-fold serially diluted and used as qPCR templates. The linear regression equations and R^2^ are shown.

**Table S1: Characteristics of gene annotation of assembled unigenes against the Nr dataset.** E-value and similarity distribution of BLASTX hits for unigenes with a cut-off E-value of 1E-5.

**Table S2:** **Screen of differentially expressed genes by pairwise comparison during anuran metamorphosis.** The results are presented in Figure 2A, B.

**Table S3: Data matrix of** **differentially expressed genes of three clusters using hierarchical clustering.** The three main clusters are colored red, green and blue. The results are presented in Figure 2c.

**Table S4: GO enriched processes in three key developmental stages.** The significantly overrepresented (FDR < 0.05) enrichment factors of GO biological processes in the three clusters are shown. Only groups containing more than five genes and having a fold enrichment factor > 3 are presented in Figure 4.

**Table S5: The expression patterns of differentially expressed genes during the three key developmental stages.** The expression count data were calculated as log _2_^(ratios)^. The list of expression patterns is presented in Figure 5.

**Table S6: Genes showing PM-specific expression**

**Table S7: Genes showing MC-specific expression**

**Table S8: Genes showing CM-specific expression**

**Table S9: Biological processes showing GO enrichment in the CM stage.** The GO biological processes showing significantly overrepresented (FDR < 0.05) enrichment factors are presented in red.

**Table S10: Time course of body size of metamorphosis in *M.fissipes* and *B.gargarizans*.** Animals were reared under identical laboratory conditions. Developmental stages were identified based on morphological characters in Gosner staging method.

**Table S11: Sequence of primers used for real-time PCR**

**Figure S1**


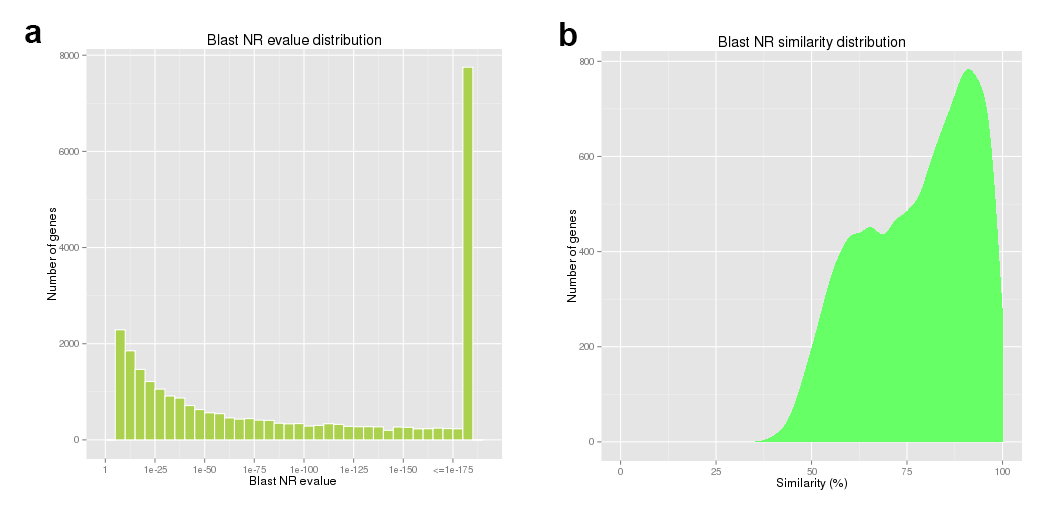


**Figure S2**


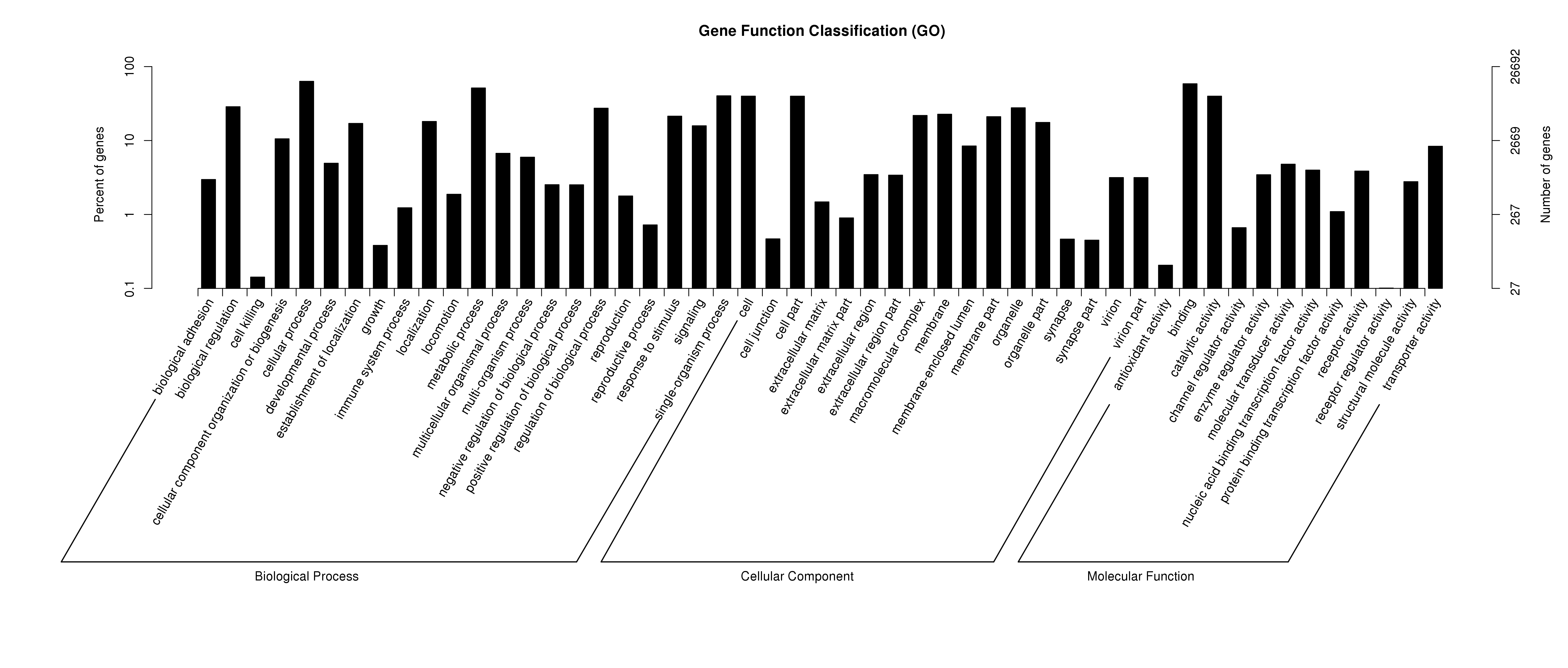


**Figure S3**


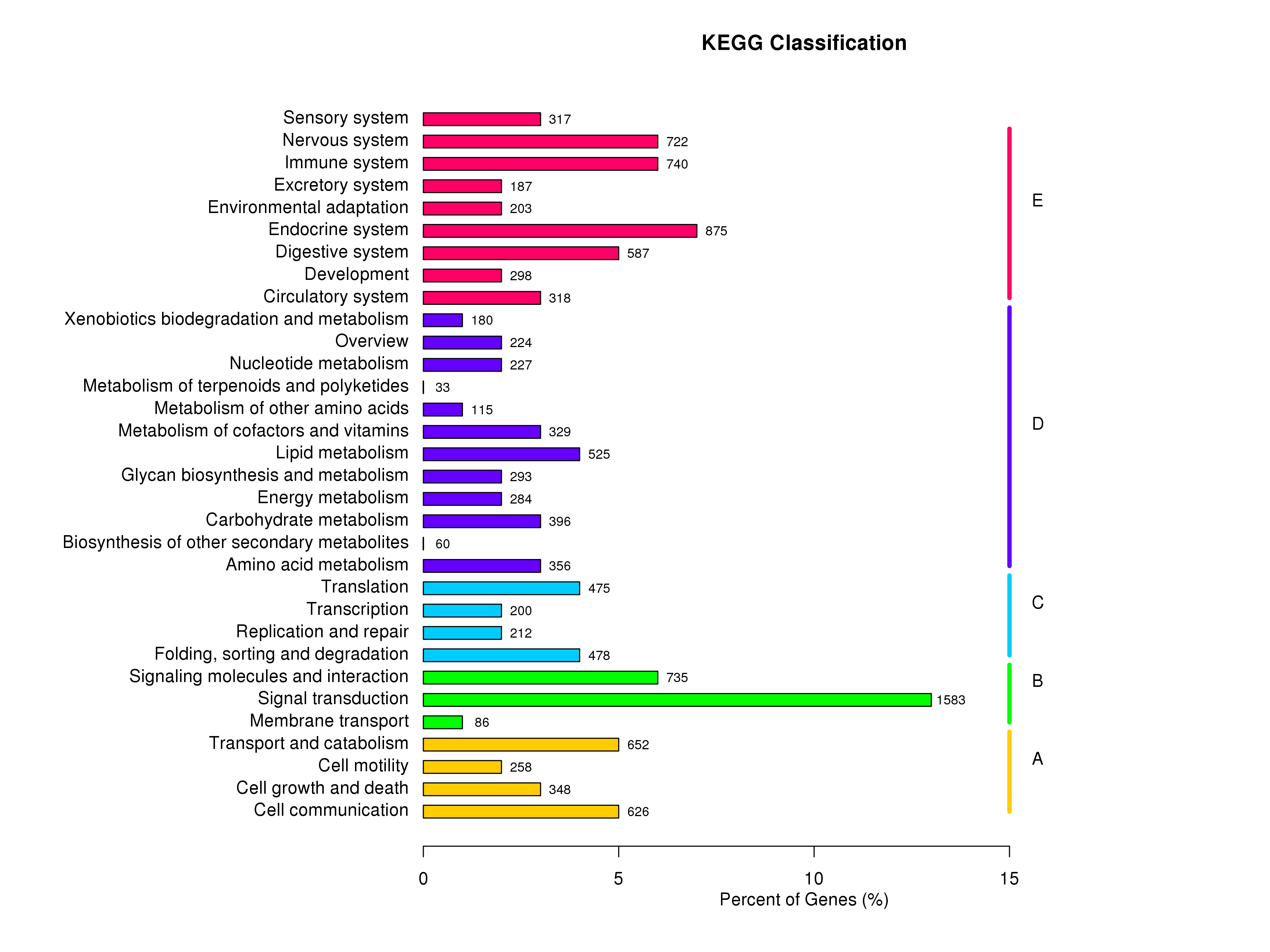


**Figure S4**


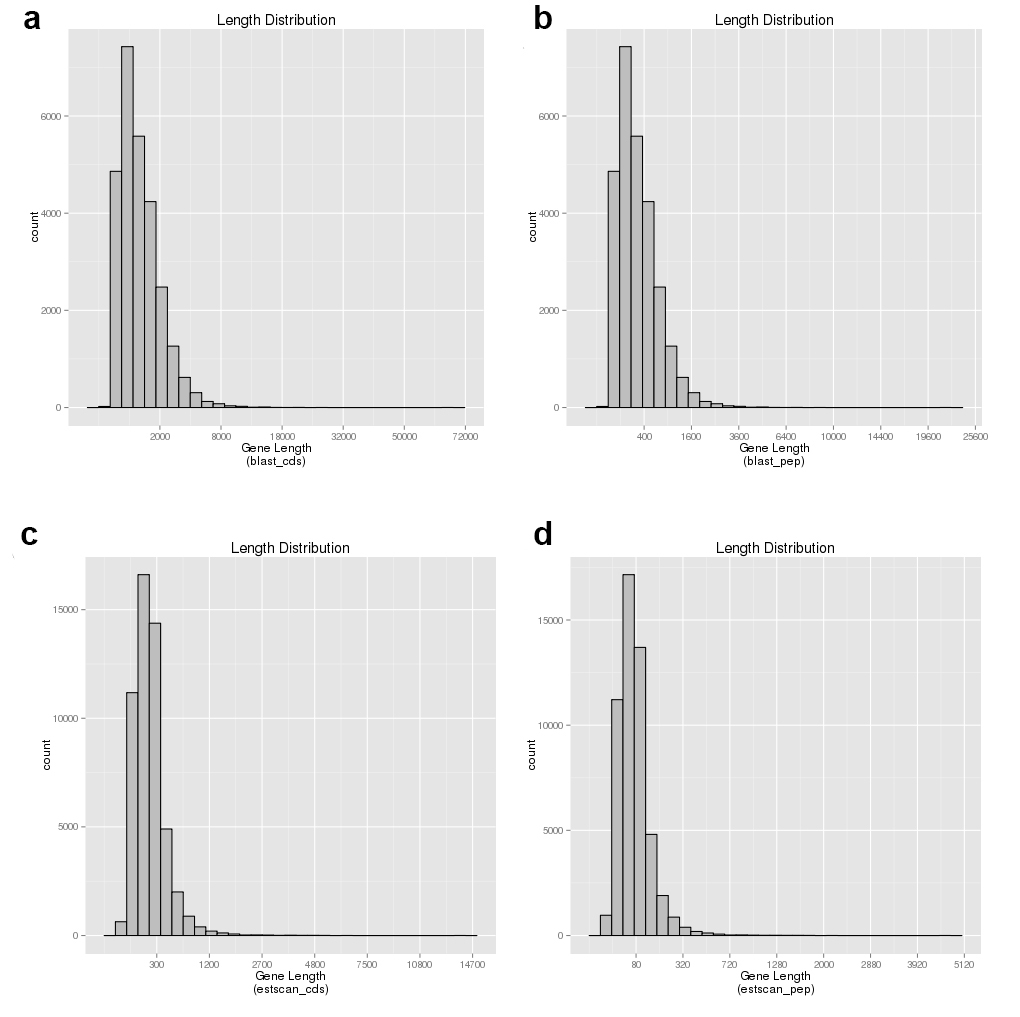


**Figure S5**


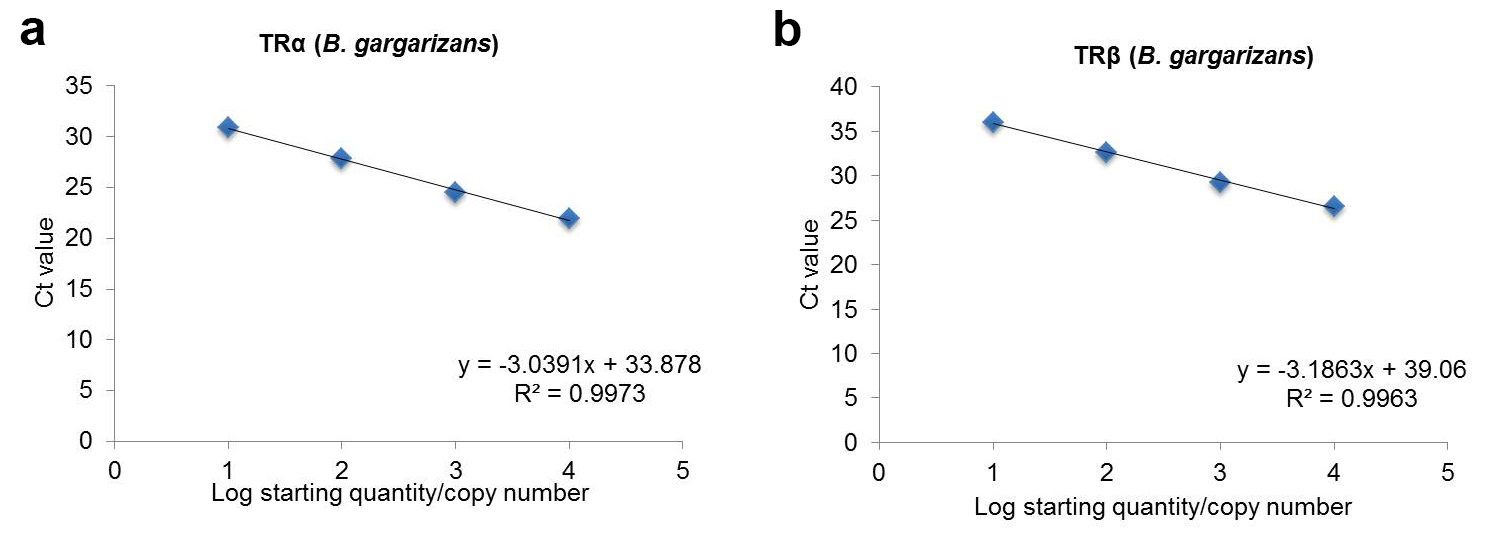


**Figure S6**

**
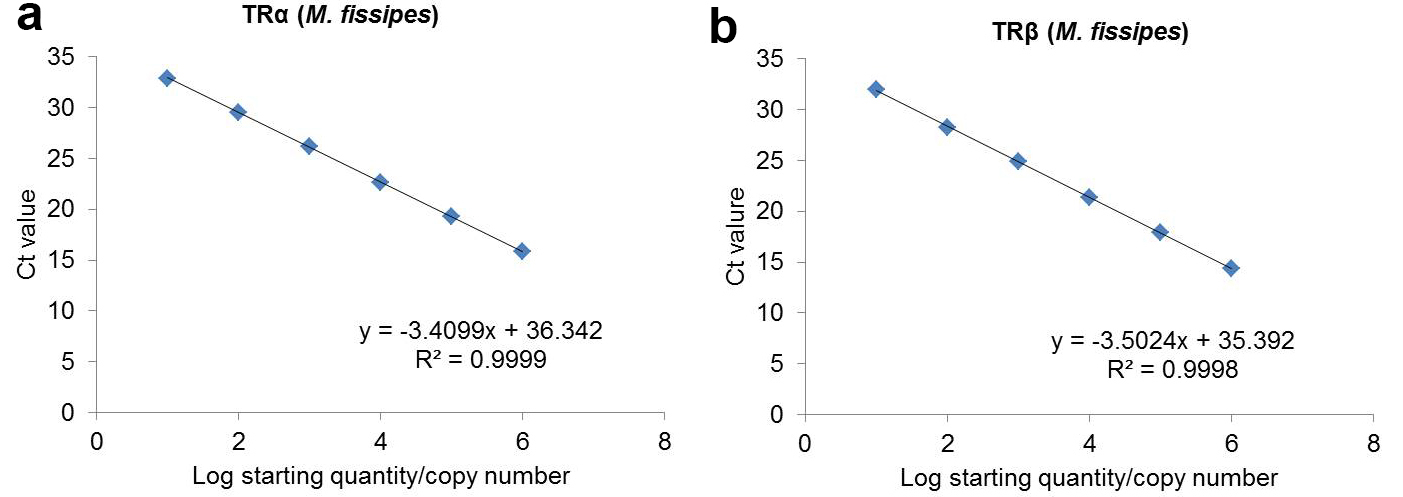
**
